# Alcohol Misuse post Metabolic and Bariatric Surgery: A Systematic Review of Longer-term Studies with Focus on new Onset Alcohol use Disorder and Differences Between Surgery Types

**DOI:** 10.1007/s13679-024-00577-w

**Published:** 2024-06-08

**Authors:** Julia S. Kenkre, Sutapa Gesell, Annalise Keller, Raffaella M. Milani, Samantha Scholtz, Elizabeth A. Barley

**Affiliations:** 1 Section of Endocrinology and Investigative Medicine, Imperial College, London, UK; 2 Central and North West London NHS Foundation Trust, London, UK; 3 School of Human and Social Sciences, University of West London, London, UK; 4 West London NHS Trust, London, UK; 5 School of Health Sciences, University of Surrey, Surrey, UK

**Keywords:** Alcohol misuse, Alcohol use disorder, Bariatric surgery, Roux-en-Y gastric bypass surgery, Sleeve gastrectomy, Alcohol use

## Abstract

**Background:**

Evidence suggests an increased risk of alcohol problems post-surgery where no problematic alcohol use was present prior to surgery which may be different across types of surgery.

**Objective:**

To characterise the risk of new onset alcohol misuse post bariatric surgery, differences between surgeries and the impact over time.

**Methods:**

All published studies on new and relapsing alcohol use were reviewed. Data were classed as ‘subjective’ (clinical interview, self-report questionnaires) and ‘objective’ (hospital admissions, substance misuse programmes) and further categorised by follow up time - ‘shorter-term’ (one year), ‘medium-term’ (one year to two years) and ‘long-term’ (> two years).

**Results:**

Twenty-three of the forty-two studies included in the review reported new onset data. Nine studies reported on differences between surgery types. In those reporting objective measures, all of which were long term, RYGB carried a higher risk than SG, followed by LAGB. All but one study using subjective measures reported a small but significant number of new onset concerning alcohol use, and comparisons between surgery types had more varied results than the objective measures. Studies of substance abuse programmes found high rates of new onset cases (17–60%).

**Conclusion:**

This systematic review provides support for the consensus guidance suggesting patients should be informed of a small but significant risk of new onset alcohol use following bariatric surgery, with the strongest evidence in the medium- to long-term and in those who have had RYGB followed by SG.

## Introduction

Bariatric surgery, of which the most common procedures worldwide are the sleeve gastrectomy (SG) (61%) followed by the Roux-en-Y gastric bypass (RYGB) (26%) [1], is the most effective treatment option for sustained weight loss, significantly reducing the morbidity and mortality associated with obesity [2]. Worldwide between 2012 and 2022, 480 970 bariatric procedures had been recorded in registries from 24 countries excluding the UK [3], where over 90,000 bariatric procedures have been recorded in the national bariatric surgery registry [4]. However, an important and under-communicated side effect is the increased risk of alcohol problems post-surgery, including in those individuals where there was no problematic alcohol use prior to surgery. It is estimated that globally around 1% of the population has an alcohol use disorder. Depending on the country, this ranges from around 0.5 to 5% [5]. The most recent studies of harmful alcohol consumption (>14 units a week) in people living with obesity, suggest the prevalence is similar to the general population, at around 1-3% [6]. Studies examining the relationship between body mass index (BMI) and alcohol misuse in large population cohorts have tended to find either no relationship or a negative relationship of alcohol intake and BMI, suggesting that obesity itself may not carry a higher risk of alcohol dependency. The risk of the development of alcohol misuse post-surgery is therefore a concern as it suggests a possible harm arising after surgery. This was originally addressed by the American Society for Metabolic and Bariatric Surgery Clinical Issues Committee [7] in their position statement in 2015 and this was followed by recommendations from the European Association for the Study of Obesity [8] and British Obesity Metabolic Surgery Society [9] recommendations to screen for alcohol use as a risk factor before surgery.

A systematic review of fifty-eight studies in 2019 [10], found that a significant portion of people who had had bariatric surgery increased alcohol intake after surgery, especially in those who had used a history of alcohol or other substance use disorder but there was also some evidence of emerging new onset alcohol use disorder in some patients. Most patients in this review underwent RYGB surgery, making it difficult to draw conclusions regarding the risk in other procedures such as SG. Due to limited evidence of the effect of SG on alcohol use, clinicians may assume that it is associated with a lower risk than RYGB; understanding this risk is of particular importance since SG is becoming an increasingly common procedure. Furthermore, methodological variations in the approach to alcohol use post-bariatric surgery, for example differences in assessment, study duration, terminology and definitions limit interpretation of data relating to new onset alcohol misuse. For instance, a recent systematic review and meta-analysis found that only 6 of the 18 studies included used well-defined metrics that made it possible to run a meta-analysis, and this found no statistically significant difference in alcohol consumption before and after bariatric surgery. There was insufficient data on the patterns of use of alcohol to draw conclusions, although the systematic review found higher alcohol use in longer term follow up [11]. The current review therefore aims to provide an updated review of this area, including more recent studies that evaluate differences between types of surgery such as RYGB and SG and emerging data on new onset of alcohol misuse.

## Methods

The protocol for this review is published on PROSPERO (https://www.crd.york.ac.uk/prospero/display_record.php?RecordID=77118). Since its publication, the focus of the review was changed to new onset alcohol use disorder post-surgery and differences between surgery types, following the publication of studies of sleeve gastrectomy.

### Eligibility Criteria

Since studies tend to include both new and relapsing alcohol use, all published studies on this topic were reviewed and data on both extracted. To be included, quantitative studies of any design needed to i) be of adult (≥18 years) participants who had undergone all forms of bariatric surgery including, but not confined to RYGB, laparoscopic adjustable gastric banding (LAGB), SG, biliopancreatic diversion (BPD), and the BPD with duodenal switch (BPD-DS); ii) studies with a follow up period of 6 months or more after surgery were included; iii) be published in English in a peer reviewed journal.

### Search Strategy and Study Selection

We searched The Cochrane Library, MEDLINE, EMBASE, SCOPUS, CINAHL and Psych INFO from inception to May 2023. We also searched trials databases: ClinicalTrials.gov (http://clinicaltrials.gov/), Register of Controlled Trials (http://www.controlled-trials.com/mrct/), the EU Clinical Trials register (https://www.clinicaltrialsregister.eu/) and the World Health Organization (WHO) International Clinical Trials Registry Platform Search Portal (http://apps.who.int/trialsearch/). A detailed search strategy is available from authors.

Three review authors (JSK, RM, SG) independently scanned the abstract and title of identified articles. Potentially relevant articles were retrieved as full text. The eligibility of full texts was assessed independently by three reviewers (JSK, RM, SS) with discrepancies resolved through discussion or recourse to the third review author (SS).

### Data Extraction and Quality Assessment

For each included study, three review authors (JSK, RM, SG) independently extracted study, participant characteristics and outcome data (alcohol use pre and post-operatively, however it had been measured) using a customised data extraction template. During this process, where available, the authors focussed on distinguishing data concerning new onset *versus* relapsing alcohol misuse as well as the type of surgery performed. Missing data were sought by emailing authors; we did not receive any response. For duplicate reports of a primary study, we used the most complete dataset aggregated across publications.

Three reviewers (MA, TA and RM) independently assessed study quality using the integrated quality criteria for review of multiple study designs (ICROMS), which uses different cut off points according to study design. The tool consists of two parts: 1) a list of quality criteria specific for each study design, as well as criteria applicable across all study designs by using a scoring system; 2) a 'decision matrix', which specifies the robustness of the study by identifying minimum requirements according to the study type and the relevance of the study to the review question [12].

### Data Synthesis

Due to the heterogeneity of study design among the included studies, a narrative synthesis was performed. Since the methods of measuring alcohol use may influence findings, data which were categorised as ‘subjective’ i.e. clinical interview and self-report questionnaires, were collated and compared with data categorised as ‘objective’ i.e. hospital admissions, admissions to alcohol or substance misuse programmes and International Classification of Diseases (ICD) and Diagnostic and Statistical Manual of Mental Disorders (DSM) alcohol related diagnostic coding. To test the impact of time, data were further categorised as ‘shorter-term’ ≤ 1 year, ‘medium term’ = 1 year to 2 years, ‘long term’ >2 years. Subjective and objective data were stratified by follow up time and then divided into new onset *versus* relapsing alcohol misuse data; the quality of evidence for each dataset was also reported with emphasis given to studies in the top tercile of ICROMS scores.

The definition of ´harmful alcohol use´ most widely used is the WHOs ICD–10 [13]:

*a pattern of psychoactive substance use that is causing damage to health. The damage may be physical (e.g. hepatitis) or mental (e.g. depressive episodes secondary to heavy alcohol intake). Harmful use commonly, but not invariably, has adverse social consequences; social consequences in themselves, however, are not sufficient to justify a diagnosis of harmful use.*

In ICD–10 alcohol dependence syndrome (AD) is defined as:

*a cluster of behavioural, cognitive, and physiological phenomena that develop after repeated substance use and that typically include a strong desire to take the drug, difficulties in controlling its use, persisting in its use despite harmful consequences, a higher priority given to drug use than to other activities and obligations, increased tolerance, and sometimes a physical withdrawal state.*

The other widely used classification system, DSM–IV, described two distinct disorders, alcohol abuse (AA) and alcohol dependence (AD), with AA being equivalent to ICD-10´s ´harmful alcohol use´, and AD being equivalent to ICD10s alcohol dependence syndrome, although more emphasis is places on the harmful consequences of use in the DSM IV. DSM–5 integrates the two DSM–IV disorders, AA and AD, into a single disorder called alcohol use disorder (AUD) with mild, moderate, and severe sub-classifications.

In this study, there are various tools described which attempt to classify alcohol use behaviours according to the ICD 10 and DSM IV or 5 diagnostic criteria outlined above. These include the Alcohol use identification test (AUDIT [14]) (cut off scores for moderate risk alcohol use disorder (MR) or AUD are ≥ 8, and severe AUD or alcohol dependency (AD) ≥ 15), Alcohol use identification test shortened version (AUDIT-C [15] )(AUD ≥ 2 for adolescents and in >18 years, ≥ 4 women, ≥ 3 women) and The Michigan Alcohol Screening Test [16] (MAST) (AD ≥ 5 ). Of these, the AUDIT is the preferred tool and was developed by the WHO as a simple screening assessment that is intended for widespread use. The AUDIT-C is a modified shortened version, not intended for diagnostic purposes and the MAST is an older tool, and not as widely used as the former two tools. In some studies DSM IV, DSM V and ICD criteria were identified by diagnostic interview.

In order to interpretate the results of this review more clearly, although AA, AD, MR and AUD are all listed separately, these categories should all be interpreted as concerning use of alcohol [17].

Where reported, alcohol use (AU) and subjective problematic alcohol use (PAU) by self-report (not according to universal criteria) was also extracted. The impact of potential risk factors, including type of surgery, length of follow up, eating disorders and other addictions, were also explored where data were available.

## Results

### Characteristics of Included Studies

**Fig. 1:**
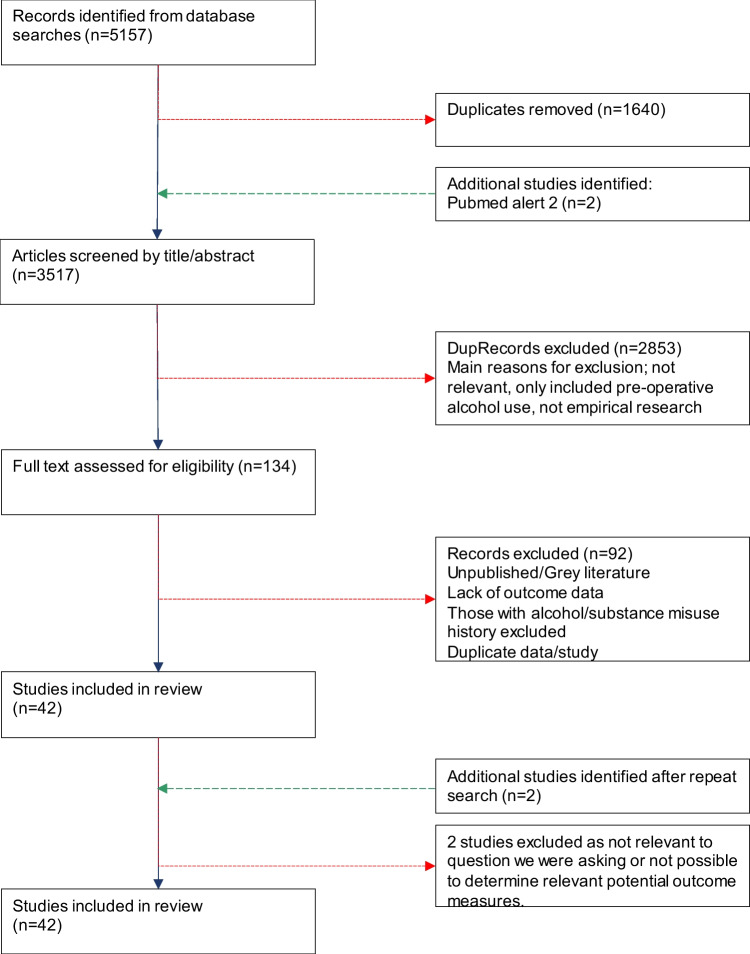
Study identification and inclusion flow diagram

We identified 5157 articles. After duplicates (n = 1640) were removed 3517 titles and abstracts were screened, of these 134 were potentially eligible and their full texts reviewed. After additional searches, 42 studies were included in this review. Figure 1 details the study selection process.

The 42 studies included data on 762,362 participants covering 3,669,656 patient years of follow up. Characteristics of the included studies, including country, design, baseline participant characteristics, alcohol use measure, length of follow up, are shown in Table 1.

**Table 1:** Baseline characteristics of included studies

**Author** **Year** **Country**	**Setting**	**Part** **(n)**	**Age (yrs)** **Mean (SD)** **(unless otherwise stated)**	**Gender** **(F/M)**	**Pre-op BMI (kg/m2)** **Mean (SD)** **(unless otherwise stated)**	**Type of surgery**	**Follow up period**
Adams et al. 2012 [18] USA	Veterans association	61	48 ± 7	33% 67%	46 ± 6	LAGB RYGB	Pre-surgery & 6,12,24 months post-op
Alfonsson et al. 2014 [19] Sweden	University hospital	129	43 ± 11	78% 22%	43 ± 4	RYGB	5 months pre-surgery & 12 months post-op
Backman et al. 2016 [20] Sweden	Hospital admissions	16755	* 18–38 45% 40–49 32% ≥ 50 23%	76% 24%	NR	RYGB	Median follow up 1.9 years (IQR 0.83–3.76)
Bhatti et al. 2016 [21] Canada	Emergency department	8815	* 18–34 20% 35–65 80%	81% 19%	NR	RYGB	3 yrs pre-surgery & up to 3yrs post-op
Bramming et al. 2020 [22] Denmark	Danish National Patient Register	13430	40 ± 10	77% 23%	41 ± 5	RYGB SG LAGB	Median 7 years, 5yrs pre-op and up to 10yrs post-op
Buffington 2007 [23] USA, Northern Europe, Israel	100 practices	318	NR	94% 6%	****** 41–50 50% > 50 or > 60 43%	RYGB Other	≥ 1 year post-op
Burgos et al. 2015 [24] Portugal	Outpatient clinic	276	42 ± 11	90% 10%	******* 115 ± 16	LAGB RYGB	Pre-op & 6,12- & 24-months post-op
Coluzzi et al. 2019 [25] Italy	Not specified	142	43 ± 11	71% 29%	43 ± 5	SG	4–6 wks pre-op & 1,3,6 & 12 months post-op
Conason et al. 2013 [26] USA	Major urban community hospital	155	40 ± 11	85% 15%	46 ± 7	RYGB LAGB	Pre-op & 1,3,6,12 and 24 months post-op
Cuellar-Barboza et al. 2015 [27] USA	Mayo Clinic Addiction Treatment Programme	41	** 46 ± 1.3	90% 10%	******** 30 ± 1	RYGB LAGB	Up to 8 yrs post-op
de Amorim et al. 2015 [28] Brazil	Surgery Clinic of the Hospital	119	41 ± 11	83% 17%	49 ± 9	RYGB	Pre-op & up to 18 months post-op
Ertelt et al. 2008 [29] USA	Hospital	70	50 ± 9	86% 14%	52	RYGB	6–10 yrs post-op
Fowler et al. 2014 [30] USA	De-identified database	154	49 ± 11	88% 12%	32 ± 7	RYGB	Mean 2.7 ± 2.2 yrs post-op
Gribsholt et al. 2018 [31] Denmark	Danish National Patient Registry	9895	42	80% 20%	********* 46 (43-51)	RYGB	Up to 4 yrs post-op
Hilgendorf et al. 2018 [32] USA	Hospital	179	46	85% 15%	48	RYGB SG	Pre-op & 6,12,18 & 24 months post-op
Ibrahim et al. 2019 [33] USA	State-wide quality collaborative (multiple institutions)	5724	*** 46 (IQR 38–56)	78% 22%	********* 46 (IQR 42–52)	RYGB SG	Pre-op & 1&2 yrs post-op
Kim et al. 2022 [34] USA	IMS Pharmetrics database	48997	45 (IQR 37–53)	79% 21%	NR	RYGB LAGB SG	At least 1 yr pre-op Median 2.8 yrs (IQR 1.8–4.2)
King et al. 2017 [35] USA	Ten US hospitals	2458	**** 47	79% 21%	********** 46	RYGB LAGB	Pre-op & yrly up to 7 yrs post-op
Krogh 2020 [36] USA	UCLA Health and the Kaighan databases	212	47 ± 10.9	72% 28%	46 ± 9	RYGB SG LAGB	Mean 4.5 ± 5.3 yrs
Kovacs et al. 2017 [37] Denmark	National Danish Psychiatric Central Research Register, Danish Register of deaths, Danish National patient register	22451	42 ± 11	75% 25%	NR	Bariatric surgery not specified	Mean 4.03 ± 2.02 yrs post-op
Lent et al. 2013 [38] USA	Large rural health system	899	50 ± 11	81% 19%	46 ± 7	RYGB	6–12 months pre-op & ≥ 365 days post-op
Mahmud et al. 2022 [39] USA	US Veterans Health Administration (VHA) Centres	6330	***** 53 (44-61)	68% 32% 32%	********* 43 (38.8–48.0) 43 (38.8–48.0)	RYGBRYGB SG LAGB	Median (IQR) 5 (3-5) yrs
McGrice and Porter 2012 [40] Australia	3 bariatric centres	52	45 ± 11	73% 27%	******* 128 ± 30	LAGB	1 yr post-op
Miller-Matero et al. 2021 [41] USA	A single institution- not specified	564	46 ± 10	84% 16%	48.1 (7.8)	RYGB SG	< 1 yr 1–2 yrs 2–3 yrs or 3–4 yrs post-op
Murray et al. 2019 [42] USA	Not specified	27	33 ± 8	93% 7%	NR	RYGB SG	4- & 24-months post- intervention
Ostlund et al. 2013 [43] Sweden	Hospitals (National Register)	11115	40 ± 10	77% 23%	NR	RYGB Restrictive	Mean 8.6yrs post-op
Reaves et al. 2019 [44] UK	Bariatric surgery support groups	14	52	64% 36%	NR	RYGB SG Other	Mean 5–9 yrs post-op
Reslan et al. 2014 [45] USA	Outpatient clinics	141	53 ± 10	79% 21%	NR	RYGB	** ≥ **24 months post-op
Saules et al. 2010 [46] USA	Substance abuse treatment facility	54	45 ± 9	38% 62%	NR	Bariatric surgery not specified	Retrospective review-not specified
Sen et al. 2021 [47]	Private bariatric centres	183	40 ± 11	63% 37%	42.7 ± 6.5	SG	Pre-op & 3.5 ± 1.6 yrs post-op
Slotman et al. (BOLD study) 2013–2017 [48, 49] USA	Registry of bariatric surgery	~600000	47 ± 12	79% 21%	****** < 35 2% 35–39.9 17% 40–49.9 54% 50–59.9 21% ≥ 60 6%	All types bariatric procedures recorded	Pre-surgery & yearly for a minimum of five yrs post-op
Smith et al. 2017 [50] USA	Post-op outpatient clinic	26	44 ± 11	85% 15%	NR	RYGB SG	1-4yrs post-op
Spadola et al. 2017 [51] USA	Not specified	69	***** 26 (Range 16–36)	75% 25%	NR	RYGB SG LAGB	Mean 19.9 months post-op (range 5–55 months)
Suzuki et al. 2012 [52] USA	Psychiatric department	51	51 ± 9	86% 14%	49 ± 8	RYGB LAGB	Mean 43.4 (SD = 6.8) months post-op
Svensson et al. 2017 [53] Sweden	25 surgical & 480 primary health care centres	2010	46 ± 6	VBG 70.9% 29.1% Banding 69.1% 30.9% GB 71.3% 28.7%	41 ± 4	VBG LAGB RYGB	8–22 yrs post-op
Strommen et al. 2021 [54] Norway	3 hospitals	546	40 ± 9	80% 20%	46.5 ± 5.6	RYGB	Mean 12 ± 1 yrs post-op
Thereaux et al. 2019 [55] France	France National Health Insurance Database	8966	40 ± 11	82% 18%	****** 30–39.9 20% 40–49.9 64% > 50 11%	RYGB SG	Mean 6.8 ± 0.2 yrs post-op
Vangoitsenhoven et al. 2016 [56] Belgium	University hospital	23	49 ± 21	74% 16%	43 ± 5	RYGB	7 yrs post-op
Wee et al. 2014 [57] USA	2 academic WLS centres	541	44	76% 24%	47	LAGB RYGB SG/other	1 & 2 yrs post-op
White et al. 2022 [58] USA	5 bariatric surgery centres in USA	217	***** 17 (15–18)	76% 24%	********* 51 (45.4–58.9)	RYGB SG	Pre-op, 6 months and up to 8yrs post-op
Wiedemann et al. 2013 [59] USA	Brighton Hospital, drug & alcohol treatment programme	56	45 ± 10	72% 28%	31 ± 7	RYGB	Retrospective review
Wong et al. 2022 [60] USA	Academic Centre	97	45 ± 12	72% 28%	********* 44.1 (41.4–48.1)	SG	Pre-op & 1 yr post-op

*LAGB* laparoscopic adjustable gastric band, *RYGB* Roux-en-Y gastric bypass, *SG* Sleeve Gastrectomy, *VBG* Vertical banded gastroplasty, *F* Female, *M* Male, *NR* not reported

*Age range (percentage); **Mean age (SEM); ***Mean age (IQR); ****Median age; *****Median (Range or IQR); ******BMI range (%); *******Mean weight in kg(SD); ********Mean BMI(SEM); *********Median BMI(IQR); **********Median BMI

### Studies Reporting Subjective Data

#### Clinical Interview Data

Nine studies reported clinical interview data (total n = 601,384) (Table 2). Overall, the quality of the studies was good, with three out of nine studies scoring equal or above the ICROMS cut-off point, and the remaining scoring just below. Four studies reported shorter term follow up, three of these reported both shorter and medium term follow up, five studies reported medium term follow and five studies reported long term follow up. One study was reported in several papers and the findings are summarised. Studies were grouped according to their follow up period.

**Table 2:** Clinical interview data by follow-up

**Follow-up**	**Study/ Measurement method**	**Follow up period**	**Pre-surgery**	Post-surgery*	**New onset**	ICROMS (cut off point)**
**Shorter term** **(1 year)**	Wee et al. [57] AUDIT-C administered during a clinical interview, to identify high-risk drinking (AUD)	1 year	AUD 17% (CI 13–21%)	AUD 13% (CI 10–17%)	AUD 7%	26 (22)
	BOLD registry, multiple studies 2013–2017 [48, 49] Registry of patient information Alcohol use/intake by self-report alcohol use (AU) – some studies report worsening (↑)	6 months 1 year	RYGB: AU 10–19% DS: AU NR LAGB: AU 9%	RYGB: AU 7–15% DS: AU 20–28% LAGB: AU NR RYGB: AU 14–16% (16.7% ↑) DS: AU 15–43% LAGB: AU NR (11.1% ↑)	NR	21 (22)
	Wong et al. [60] AUDIT-C to identify high-risk drinking (AUD)	1 year	SG: AUD 13.4%	SG: AUD 22.7%	AUD 19%	20 (22)
	White et al. [58] AUDIT-C to identify potential hazardous drinking (AUD) and. AUDIT to identify AU	1 year	RYGB: AU 8% AUD 2.6% SG: AU 8% AUD 4.8%	RYGB: AU 12% AUD 2.9% SG: AU 24% AUD 3.6%	NR	24 (22)
**Medium term** **(1–2 years)**	Wee et al. [57] AUDIT-C administered during a clinical interview, to identify high-risk drinking (AUD)	2 years	AUD 15% (CI 10–17%)	AUD 13% (CI 10–17%)	AUD 6%	26 (22)
	BOLD registry, multiple studies 2013–2017 Registry of patient information Alcohol use/intake by self-report alcohol use (AU) – some studies report worsening (↑)	18 months 2 years	RYGB: AU 10–19% DS: AU NR LAGB: AU 9%	RYGB: AU 12–16% DS: AU 25–44% LAGB: AU NR RYGB: AU 0–17% DS: AU NR LAGB: AU NR	NR	21 (22)
	Spadola et al. [51] Survey of sample from another study with open ended questions Alcohol abuse (AA) and Alcohol Dependence (AD) using structured clinical interview from DSM disorders I -Research Version/Non-patient edition, DMS-IV and alcohol use chart	19 months (5-55 months)	AA 15% AD 6%	AA 15% AD 0% 15% binge drinking	AA 4% AD 0%	20 (22)
	White et al. [58] AUDIT-C to identify potential hazardous drinking (AUD) and. AUDIT to identify AU	2 years	RYGB: AU 8% AUD 2.6% SG: AU 8% AUD 4.8%	RYGB AU 26% AUD 6.8% SG AU 39% AUD 12.1%	NR	24 (22)
	Sen et al. [47] AUDIT to identify AUD (≥ 8) and AD ≥ 20	1–3 years	SG: AUD 11.5% AD 0%	SG AUD 5.9% AD 1.9%	NR	21 (22)
**Long term** **(> 2 years)**	Reaves et al. [44] Retrospective semi-structured interview developed for the study to identify problematic alcohol use (PAU)	8 years	PAU 14%	PAU 42%	PAU 29%	26 (22)**
	Suzuki et al. [52] Structured clinical interview with diagnosis of AUD based on DSM-V	3 years	AUD 0%	AUD 11.8%	AUD 2%	18 (16)**
	White et al. [58] AUDIT-C to identify potential hazardous drinking (AUD) and AUDIT to identify AU	8 years	RYGB: AU 8% AUD 2.6% SG: AU 8% AUD 4.8%	RYGB AU 69% AUD 22.5% SG AU 70% AUD 32.2%	NR	24 (22)
	Sen et al. [47] AUDIT to identify AUD (≥ 8) and AD ≥ 20	4–6 years	SG: AUD 11.5% AD 0%	SG AUD 17.3% AD 6.1%	AD 5%	21 (22)
	Krogh et al. [36] Interview as part of larger data collection and during clinical interview AUDIT to identify AUD and AUDIT-C plus alcohol counselling or hospitalisation to identify AUD^a^	0–29 years (mean 4.5 years)	NR	AUD 6.1% RYGB > SG > LAGB AUD^a^ using AUDIT-C 22% SG > RYGB > LAGB	NR	20 (18)

AUDIT C cut off for hazardous drinking (AUD) ≥ 2 for adolescents > 18 years, ≥ 4 women, ≥ 3 women

AUDIT cut off for hazardous drinking (AUD) ≥ 8, for moderate severe AUD or AD ≥ 15

*AA* alcohol abuse, *AD* alcohol dependency, *AU* alcohol use, *AUD* alcohol use disorder, *PAU* problematic alcohol use

*Statistically significant increase; **Equal or above the cut-off point

^a^AUDIT C plus history of alcohol counselling or hospitalisation

##### Shorter and Medium Term Follow Up

Short- and medium-term studies depicted mixed results. Of the studies with the highest ICROM scores, Wee showed new onset AUD of 7% at 1 year and 6% at 2 years in mixed surgery group [57]. White found increased AU in both RYGB (12%) and SG (24%) at 1 year which further increased at 2 years (RYGB 26% and SG 39%) and increased AUD at year 2 (RYGB 6.8% and SG 12.1%) [58]. Studies utilising the Bariatric Outcomes Longitudinal Database (BOLD) [48, 49] did not measure AUD and found little change in AU in RYGB or LAGB both in the short and longer term follow up. Wong examined patients who underwent SG and found an increase in AUD from 13 to 22% at 1 year, and new onset AUD 19% [60]. Spadola found new onset AUD 1–2 years (19-month average) after surgery at 4%, whereas Sen found a small decrease in overall AUD rates [51].

##### Long Term Follow Up

Five studies measured prevalence of AA, AD or AUD using structured clinical interview according to the Diagnostic and Statistical Manual of Mental Disorders (DSM) criteria of the time. Two studies scored equal or above the ICROMS cut-off. Sen found rates of new onset AD to be 5% of the sample and no reported AUD at 4–6 years follow up, using AUDIT criteria [47]; Suzuki et al. found 2% of their sample had new onset AUD at 3 years follow up, using DSM V criteria [52]. Reaves et al. purposefully recruited patients reporting PAU and so was not designed to measure prevalence [44]. In this group, 29% of the fourteen participants interviewed reported that PAU had its onset after bariatric surgery i.e. new onset. PAU was defined as subjective assessment by the participant or others (medical professional) as being hazardous or requiring assistance or support, difficulty controlling alcohol use, causing concern or guilt, having a prominent role in their lives, and not having made an effort to discontinue alcohol intake. The study had the longest average follow up period of 8 years, and the smallest number of participants as it was a qualitative study.

Sen found increases in AUD in SG patients between 11.5 to 17.3% and AD 0% to 6.1% post operatively [47]. Suzuki found increase in AUD from 0% to 11.8% post operatively, type of surgery not specified [52]. In an 8 year follow study, using AUDIT-C criteria, White found AUD increased from 2.6% to 22.5% in RYGB and 4.8% to 32.2% in SG [58]. Krogh measured post-operative prevalence only in their dissertation study and found 6.1% AUD using AUDIT at a mean 4.5 year follow up, with RYGB making up the highest proportion of these cases, followed by SG and then LAGB [36].

In summary, there is evidence for new onset AUD in post bariatric patients at a prevalence between 5 and 7%, mainly after 2 years. There is an increase in AUD between pre and post op patients in all surgical groups, but more prevalent in RYGB and SG. The prevalence of post-operative AUD appears to increase over longer follow up periods. When comparing between RYGB and SG, data is mixed. However, SG is at least similar, and in some studies higher in prevalence of AUD compared to RYGB for long-term AUD.

#### Self-report Questionnaires

Twenty one studies (total n = 13,174) reported questionnaire data (see Table 3). Studies employed a range of measures of alcohol use of concern. Most studies used validated questionnaires including AUDIT (seven studies), AUDIT-C (four studies) and MAST (three studies) or questionnaires where alcohol use was captured as part of a wider data collection (seven studies). Three studies of the 21 used DSM-IV or DSM-V criteria as the basis for questionnaires. Twelve of the studies were considered high quality according to ICROMS scores. Seven studies reported shorter term, eight reported medium term and nine reported long term follow up periods. Two studies reported both short and medium term follow up and one study short and long term follow up.

**Table 3:** Self-report questionnaires

**Follow-up**	**Study/ measure of alcohol use**	**Follow up period**	**Pre-surgery**	Post-surgery*	**New onset**	ICROMS** score (cut off)
**Short term** **(up to 1 year)**	Alfonsson et al. 2014 [19] AUDIT > 8 high-risk alcohol use disorder (AUD) or > 16 likely dependence (AD)	1 year	AUD 13.9% AD 0%	AUD 5.4% AD 2.3%	AUD NR AD 2.3%	24 (22)**
	Coluzzi et al. 2019 [25] AUDIT > 8 high-risk alcohol use disorder (AUD)	1 year	AUD 4.2%	AUD 0%	NR	24 (22)**
	Lent et al. 2013 [38] Retrospective self- reported survey of alcohol use (AU) and high-risk use (≥ 5 units) on typical drinking occasions (HR)	1 year	AU 72.3% HR 7.7%	AU 62.3%* HR 3.2%	AU 6.4%	17 (18)
	McGrice and Porter 2012 [40] Cancer Council of Victoria Food Frequency Questionnaire – alcohol use (AU) in grams/d	1 year	NR	Mean AU 4.8 ± 7.5 g HR 3.8%	NR	16 (18)
	Conason et al. 2013 [26] Compulsive Behaviors Questionnaire measures alcohol use (AU) and problematic alcohol use (PAU)	1 year	AU 61.3% PAU 15%	AU 20.2% PAU 13%	NR	22 (22)**
	Ibrahim et al. 2019 [33] AUDIT-C to identify alcohol use disorder (AUD)	12 months	AUD 9.6% RYGB: AUD 7.6% SG: AUD 10.1%	AUD 8.5% RYGB: AUD 9% SG: AUD 6.3% (SG)	RYGB: AUD 0.54% SG: AUD 0.75%	25 (22)**
	Miller-Matero et al. 2021 [41] Online survey measuring self-reported AU and AUDIT-C	< 1 year	2.5% AUD	AU 43.2% AUD 2.0%	NR	17 (18)
**Medium term** **(> 1–2 years)**	deAmorin et al. 2015 [28] AUDIT-C to measure alcohol use (AU) alcohol use disorder (AUD) or likely dependence (AD)	18 months	AU 26.6% AUD 8.3% AD 0%	AU 35.1%, AUD 0% AD 3.8%	AD 3.8%	22 (22)**
	Buffington 2007 [23] Retrospective survey of self-reported problematic alcohol use (PAU)	1–2 years	PAU 4.5%	PAU 28.4%	NR	14 (18)
	Adams et al. 2012 [18] Pre-operative structured clinical interview with diagnosis of alcohol use disorder (AUD) based on DSM-V and post-operative AUDIT-C	2 years	AUD 8% (lifetime)	AUD 0%	NR	25 (22)**
	Burgos et al. 2015 [24] Self-report survey for alcohol use (AU)	2 years	AU 24.5% 2 ± 0.6 units/d	AU 9.4% 1.8 ± 0.7 units/d	AU 0%	18 (22)
	Conason et al. 2013 [26] Compulsive Behaviors Questionnaire measures alcohol use (AU) and problematic alcohol use (PAU)	2 years	AU 61.3% PAU 15%	RYGB: AU 63.2%* PAU 9%	NR	22 (22)**
	Hilgendorf et al. 2018 [32] AUDIT (scored as percentage – interpretation not clear)	6 months 12 month 18 month 24 month	0.97	0.39 0.56 0.92 0.63	NR	18 (22)
	Ibrahim et al. 2019 [33] AUDIT-C to identify alcohol use disorder (AUD)	24 months	AUD 9.6% RYGB: AUD 7.6% SG: AUD 10.1%	AUD 14% RYGB: AUD 11.9%* SG: AUD 14.4%*	RYGB: AUD 7.2%* SG: AUD 8.5%*	25 (22)**
	Murray et al. 2019 [42] Self-reported alcohol use (AU) per week	24 months	AU < 1/week	AU1-2/week*	37% AU	23 (22)**
**long term** **(> 2 years)**	Miller-Matero et al. 2021 [41] Online survey measuring self-reported AU and AUDIT-C	up to 4 years	2.5% AUD	RYGB: AU 56% AUD 15.7% SG: AU 58.8% AUD 16.3 %	7.8% AUD	17 (18)
	Smith et al. 2017 [50] **Only self-reported PAU participants recruited** Structured interview to assess DSM-IV alcohol abuse (AA) or alcohol dependency (AD) AUDIT with cut-off > 8 alcohol disorder (AUD) MAST with cut-off > 4 to indicate probable substance use disorder (SUD)	1–4 years	AA 22.7% AD 36.4%	AA 25% AD 31.8% AUD 40.9% SUD 34%	AA or AD 26% AUD 18% SUD 15%	23 (22)**
	Fowler et al. 2014 [30] MAST-AD with cut-off > 5 to indicate probable substance use disorder (SUD)	Mean 2.7 years	21.4% SUD	18.8% SUD	12. 3% SUD (surgery significantly longer ago relative to the recovered group (3.63 ± 2.41 versus 1.81 ± 1.78 years)	18 (18)**
	Ertelt et al. 2008 [29] Post-Bariatric Surgery Questionnaire – DSM IV criteria used to determine alcohol abuse (AA) or alcohol dependency (AD)	6–10 years	AA 1.4% AD 7.1%	AA 1.4% AD 8.6%	AA 0% AD 2.9%	20 (22)
	LABS-2 (35, 61)(35,61) AUDIT for alcohol use (AU) and > 8 alcohol disorder (AUD) Self-report of substance use disorder (including alcohol) treatment (SUD Rx) New onset reported as cumulative incidence	5–7 years	AU 6–8% RYGB: AUD 6.6% LAGB: AUD 7% SUD Rx 0%	AU 17%* RYGB: AUD 16.4%* LAGB: AUD 7% SUD Rx 2%	RYGB: AUD 20.8% SUD Rx 3.5% LAGB: AUD 11.3% SUD Rx 0.9%	28 (22)**
	Reslan et al. 2014 [45] MAST-AD with cut-off > 5 to indicate probably substance use disorder (SUD)	Mean 6 years	SUD 9.9%	SUD 14%	SUD 9.9%	24 (22)**
	SOS (62) (53, 63) Self-report questionnaire for alcohol use (AU), moderate risk AU according to WHO criteria (MR) and self-reported problematic alcohol use (PAU)	Median 10 years	RYGB: AU 4-10 g/d LAGB: AU 4-10 g/d MR NR PAU NR	RYGB: AU 7-15 g/d MR 7%* PAU 6% LAGB: AU 5-12 g/d MR 4% PAU 1%	NR	(Svensson, 2013) 26 (18)** (Kenerva 2017) 17 (18)
	Strommen et al. 2021 [54] Self-report questionnaire in larger study (BAROBS) with six indicator questions on AUDIT used to identify Presumed Problematic drinking behaviour (PPDB)	10–15 years (mean 12 years)	RYGB: re 2.6%	RYGB: AU 83.3% (33.3% ↑) PPBD 7.5%	NR	16 (18)
	Vangoitsenhoven et al. 2016 [56] AUDIT > 8 alcohol disorder (AUD)	7 years	NR	RYGB: AUD 13% Control: AUD 4%	NR	

*AU* alcohol use, *HR* high-risk alcohol use, *PAU* self-reported problematic alcohol use, *AUD* alcohol use disorder, *AD* likely alcohol dependency, *MR* medium risk alcohol use according to WHO criteria, *SUD* substance use disorder, *SUD* Rx substance use disorder treatment, *SCID* structured clinical interview for DSM IV axis 1 disorders, *PPDB* presumed problematic drinking behaviour, *LAGB* laparascopic adjustable gastric banding, *RYGB* Roux-en-Y gastric bypass, *SG* sleeve gastrectomy

*Statistically significant increase; **Equal or above cut off point

##### Shorter Term Follow Up

Of the seven studies reporting shorter term follow up, three reported on new onset alcohol use. Alfonsson et al. [19] reported a 2.3% prevalence of new onset AD (AUDIT cut-off >16) and Ibrahim et al. [33] reported 0.54% prevalance of AUD (AUDIT cut off >8) in RYGB patients at 12 months. New onset AU was reported in 6.4% of those completing a self-survey by Lent et al. [38]. Most studies showed unchanged or reduced prevalence of AUD in the first year after surgery.

##### Medium Term Follow Up

 Of the eight studies reporting follow up of between 1 and 2 years after surgery, four reported on new onset alcohol use. Using self-report surveys, Burgos et al. [24] ound no new onset AU, whereas Murray et al. [42] found new onset AU in 37%. De Amorin et al. [28] found new onset AD in 3.8% of RYGB patients at 18 months post-surgery (AUDIT-C cut off >8) but also reported a reduction in prevalence of AUD from 8.3% to 0%. Ibrahim et al. [33] found new onset AUD in 7.7% of RYGB and 8.5% SG patients 2 years post-surgery (AUDIT C cut off > 3 (F)/ > 4 (M)), with significant increases in AUD in RYGB patients from 7.6 to 11.9% and SG from 10.1 to 14.4% when comparing pre- to 2 year post-operative rates.

##### Long Term Follow Up

Of the nine studies reporting follow up more than 2 years post-surgery, six reported on new onset alcohol use ranging from 2.9–26% AA and 3.5–20.8% AUD or SUD. Fowler et al. [30], Smith et al. [50] and Reslan et al. [45] reported new onset SUD (including alcohol) of 12.3%, 15%, and 9.9% using MAST (cut off scores of 4 or 5, follow up between 2 and 6 years). King et al. [35] reported a cumulative incidence over 5 years of new treatment for SUD in 3.5% of RYGB patients. Using a combination of semi-structured interview against DSM-IV criteria and the AUDIT (cut-off >8) Smith et al. [50] found 26% new onset AA and 18% new onset AUD in RYGB patients at 2 year follow up, and King et al. [35] found a cumulative incidence of 20.8% new onset AUD at 5 years follow up in longitudinal data set (LABS-2). Ertelt et al. [29] found no new onset AA but 2.9% new onset AD according to a questionnaire which used DSM-IV criteria. Miller-Matero et al. [41] found new onset AUD in 7.8% at between 2–4 years after SG and RYGB surgery, using self-report of AUDIT-C. Vangoitsenhoven also reported a higher prevalence of AUD in the RYGB group as opposed to a matched controlled obese group (13% vs 4%), at 7 year follow up [56]. In summary, studies using self-reporting questionnaire showed a tendency for reduction in alcohol use and/or no significant change in prevalence of alcohol use disorder in the first year after surgery, and increases in AU, PAU and AUD 2 years and more after surgery. There was insufficient data to compare the risk between operations, and most data pertained to SG and RYGB. From existing data, both operations appear to carry a risk of increased AUD after 2 years. The three longitudinal follow up datasets report on RYGB data only and confirm this risk to be persistent, with increased AUD at five to seven years (King et al. [35]), PPDB increased to 7.5% after a mean of 12 years (Strommen et al. [54]) and MR drinking according to WHO criteria after 10 years (Svensson et al. [53]).

#### Studies Reporting Objective Data

##### ICD Diagnoses in Patient Data

Nine studies reported alcohol-related ICD data from hospital admission data or bariatric surgery registries (total n = 146,752) (see Table 4). The overall quality of the studies was good, with six out of nine scoring above the quality cut-off point (See Table 4). All nine studies reported findings at follow up greater than 2 years (mean follow up periods of 4–8 years). Seven studies examined in-patient hospital cohorts over a period of time and used ICD-8, ICD-9 or ICD-10 codes to extract AUD diagnoses during admissions and two studies extracted alcohol related ICD codes from bariatric surgery databases. Three studies compared the relative risk of having AUD during an admission in patients who had previously undergone bariatric surgery compared to matched controls (Backman et al. [20], Kovacs et al. [37] and Thereaux et al. [55]). Two studies examined the relative risk of having AUD diagnosis during admission in cohorts of patients who had undergone bariatric surgery compared to themselves in the years preceding bariatric surgery (Bhatti et al. [21], Gribsholt et al. [31]). Ostlund et al. [43] and Mahmud et al. [39] compared the relative risk of AUD diagnosis during admission between surgery types, specifically RYGB patients compared to a restrictive operation. Three studies compared different types of bariatric surgery patients (RYGB, LAGB, SG) to control population (cholecystectomy) (Kim et al. [34], Thereaux et al. [55]) or age and BMI matched controls (Bramming et al. [22]) None of the studies reported on new onset alcohol use disorder. All studies showed an increased relative risk of AUD diagnosis in bariatric surgery patients during hospital admissions, compared to matched controls, for RYGB compared to LAGB or SG, and in bariatric surgery patients comparison of pre- to post- surgery.

**Table 4:** ICD diagnosis in patient data sets

**Follow-up**	**Study/ measure of alcohol use**	**Follow up period**	**Pre-surgery**	Post-surgery*	**New onset**	ICROMS**
**Long term** **(> 2 years)**	Backman et al. 2016 [20] ICD-8, ICD 9 and ICD10 codes for AUD Incidence rate ratio (IRR) and post-operative hazard ratio (HR) of AUD in hospital admissions in bariatric surgery patients compared to matched population	4 years	AUD IRR 1.13 (1.00–1.27 compared to controls	AUD HR 2.73 (2.36–3.15)* M 2.90 (2.30–3.67)	NR	23 (18)**
	Bhatti et al. 2016 [21] ICD-8, ICD 9 and ICD10 codes for AUD Prevalence of AUD in emergency admissions for self-harm in RYGB patients 3 years post-surgery compared to 3 years pre-surgery	3 years	AUD 0.6%	AUD 3.2%	NR	28 (22)**
	Gribsholt et al. 2016 [31] ICD-10 codes for AUD Incidence rate ratio (IRR) of alcohol abuse (AUD) in hospital admissions pre-surgery and post-surgery	4 years	AUD IRR 0.59 (0.39 - 0.88)	AUD IRR 2.17 (1.72–2.72)*	NR	17 (18)
	Kovacs et al. 2017 [37] ICD-10 codes for AUD Hazard ratio (HR) of AUD in psychiatry hospital admissions post-RYGB compared to non-operated, controlling for age and gender	4 years	NR	AUD HR 3.91, (2.94–5.18)*,	NR	27 (22)**
	Mahmud et al. 2022 [39] ICD-9 and ICD-10 codes for AUD Incidence rate ratio (IRR) of AUD in hospital admissions post RYGB compared to post SG	5 years	NR	AUD IRR 2.12 (1.64–2.75)* RYGB: 24.6 AUD hospitalisations per 1000 patient-years for SG: 11.6 per 1000 patient-years	NR	16 (18)
	Ostlund et al. 2013 [43] ICD-8, ICD-9 and ICD-10 codes for AUD Incidence rate ratio (IRR) of AUD in hospital admissions post-RYGB compared to post-restrictive procedure (e.g. LAGB)	8 years	AUD IRR 1.1 (0.8 - 1.4)	AUD IRR 2.3, (1.7 - 3.2)* RYGB	NR	19 (18)**
	Kim et al. 2022 [34] ICD-9, ICD-10 AUD and alcohol related diagnoses Private healthcare database post bariatric surgery compared to cholecystectomy	3 yrs	0% AUD (study excluded patients with AUD)	RYGB 4% (AHR = 1.51, 95% CI 1.40–1.62)* LAGB 1.3% (AHR = 0.55, 95% CI 0.48–0.63) SG 1.8% (AHR = 0.77, 95% CI 0.64–0.91)	2.7% vs 1.9% controls AA 1.9% vs 1.1% AD 0.9% vs 0.6%	20 (18)**
	Bramming et al. 2020 [22] ICD-10 defined AUD alcohol-related diagnosis or registration on alcohol related treatment register HR of AUD related diagnoses in register database bariatric surgery pre- compared to post and compared to age and BMI matched control	7 year	0% AUD (study excluded patients with AUD)	HR 7.29 (95% CI: 5.60–9.48) vs 7.27 (95% CI: 5.40–9.80) Pre to post HR: 7.7 (95% CI: 6.17–9.79)] RYGB HR: 7.63 (95% CI: 5.87–9.92)] vs other bariatric 2.17 (95% CI: 1.04–4.52)	5 years AUD 3.7% vs 0.8% controls and 10 years absolute risk 7.8% vs 1.4%	20 (18)**
	Thereaux et al. 2019 [55] ICD-10 codes for AUD Incidence risk ratio for AUD in hospital admissions post- RYGB, post-SG compared to matched controls	7 years	NR	RYGB* AUD IRR 1.5 (1.1 – 2.0) SG AUD IRR 0.7 (0.4 – 1.0)	NR	16 (18)

*AU* alcohol use, *HR* high-risk alcohol use, *PAU* self-reported problematic alcohol use, *AUD* alcohol use disorder, *AD* alcohol dependency, *MR* medium risk alcohol use according to WHO criteria, *SUD* substance use disorder, *SUD* Rx substance use disorder treatment, *SCID* structured clinical interview for DSM IV axis 1 disorders, *PPBD* presumed problematic drinking behaviour, *LAGB* laparascopic adjustable gastric banding, *RYGB* Roux-en-Y gastric bypass, *SG* sleeve gastrectomy

*Statistically significant increase; **Equal or above the cut off point

#### Substance Misuse Programme Attendance

Three studies (total n = 151) retrospectively analysed medical records to report on the prevalence of previous bariatric surgery amongst substance misuse programme attendees and identified patterns of alcohol and substance use and disorder. One study (Wiedemann et al.) [59] recruited a subset of the identified cohort for further semi-structured interview and AUDIT completion. The follow up periods for the two studies where this was reported were between 1 and 7 years. All three studies scored above the ICROM cut-off score indicating that the quality was good.

The studies reported prevalence rates of previous bariatric surgery amongst programme attendees of between 2 and 6%. The time from surgery to development of substance use disorder ranged between 1.4 and 5.4 years, with the majority of admissions occurring later after surgery, i.e. more than 5 years, although admission to a substance misuse programme may not represent the first episode of AUD. Amongst programme attendees who had previously had bariatric surgery, new onset AUD was reported as 17% in Cuellar et al. study [27] (did not drink prior to surgery) and new onset PAU was reported by 38.1% of those reporting alcohol use in Saules’ study [46]. New onset SUD (including alcohol) was reported as 43.4% by Saules et al. [46] and 60% by Wiedemann et al. [59]. In Wiedemann’s study, a sample of patients who had previously undergone bariatric surgery was recruited for further clinical interview (n=51), and comparison made between those whose onset of SUD was new (60%) compared to those with previous SUD. Both groups were consuming high amounts of alcohol, but there was no difference between the groups in the amount consumed, number of drinking days or AUDIT score. Those with a new onset disorder had developed a SUD later in life (40s to 50s) than is usually seen (late teens) those with a past history of SUD were using a greater number of substances and more likely to have a diagnosis of binge eating disorder (BED) pre-surgery (Table 5).

**Table 5:** Substance Misuse Programme Attendance

**Follow-up**	**Study/ measure of alcohol use**	**Follow up period**	**Pre-surgery**	Post-surgery*	**New onset**	ICROMS** (score, cut off point)
**Long term** **(2 + years)**	Cuellar et al. 2015 [27] Clinical record review of addiction centre admissions -cohort bariatric surgery compared to matched controls Self-reported alcohol use (AU), diagnosis of AUD based on DSM-V	Mean 5 years	AUD 39% AU 2.5 ± 0.44 drinks/d (bariatric)	RYGB 4.9% of AUD sample AU 8.1 ± 1.2 drinks/d(bariatric)* AU 9.6 ± 0.5 drinks/day(controls)	AUD 17%	19 (18)**
	Saules et al. 2010 [46] Clinical record review of addiction centre admissions -cohort RYGB compared to matched controls Self-reported alcohol use (AU), problematic alcohol use (PAU) or substance (including alcohol) use (PSU), diagnosis of AUD based on coding	Mean 5 years	PAU 61.9% PSU 35.8%	RYGB 2–6% of AUD sample AU 13.1 ± 9.9 drinks/d (RYGB)* AU 9.3 ± 6.7 drinks/d (controls)	PAU 38.1% PSU 43.4%	22 (18)**
	Wiedemann et al. 2013 [59] Clinical record review of addiction centre bariatric surgery invited to interview Diagnosis of SUD based on ICD-10, AU assessed by semi-structured interview and AUDIT-R	NR (mean time to SUD 1.6 years)	SUD 40%	WLS 2.8% of SUD sample AUD 68.8% (bariatric)* AUD 54.6% (control) AU 15.97–22.51 drinks/d	SUD 60% AU 11.81- 16.94 drinks/d	23 (18)**

*AU* alcohol use, *HR* high-risk alcohol use, *PAU* self-reported problematic alcohol use, *AUD* alcohol use disorder, *AD* alcohol dependency, *MR* medium risk alcohol use according to WHO criteria, *SUD* substance use disorder, *SUD* Rx substance use disorder treatment, *SCID* structured clinical interview for DSM IV axis 1 disorders, *PPBD* presumed problematic drinking behaviour, *LAGB* laparascopic adjustable gastric banding, *RYGB* Roux-en-Y gastric bypass, *SG* sleeve gastrectomy

*Statistically significant increase; **Equal of above cut off point

## Discussion

### Principal Findings

We found 42 studies reporting alcohol use post-bariatric surgery. Nine studies compared RYGB to other bariatric surgeries, mainly SG and LAGB. Of these nine studies, five studies (Bramming et al. [22], Kim et al. [34], Mahmud et al. [39], Ostland et al. [43], Thereaux et al. [55]) extracted alcohol related ICD codes from bariatric databases or hospital admission databases. These studies are of particular significance in that they represent large numbers of patients, are extracted from objective data according to diagnostic coding, rather than self-reported questionnaire or interview data, and have long follow up periods. Three hospital admission studies (Mahmud et al. [39], Ostlund et al. [43], Thereaux et al. [55]) found RYGB patients to have a higher risk of admission with alcohol related ICD diagnoses compared to SG or LAGB. Two bariatric surgery database studies (Bramming et al. [22], Kim et al. [34]) also found higher alcohol related ICD diagnoses in the RYGB patients compared to SG or other controls. Three studies (White et al. [58], Krogh et al. [36], Slotman et al. [48, 49] using validated self-report questionnaires or diagnostic interview found RYGB to have higher rates of AU and AUD compared to SG, LAGB and other operations. In contrast, two other studies using AUDIT-C cutoffs (White et al. [58], Ibrahim et al. [33]) found higher rates of AUD in SG compared to RYGB at short, medium and long term time points.

Of the 42 studies included, 23 reported new onset alcohol use of concern. These studies used a range of subjective (clinical interview, questionnaires) and objective (hospital admissions, attendance on substance abuse programmes) methods. The study quality was generally good but there were a range of measures used, and differing definitions of concerning alcohol use, including AA, AUD, AD and other ways of categorising alcohol related disorders (SUD, PAU), which made interpretation across studies difficult. In studies using clinical interview, rates of new onset AUD or AD ranged between 0–7%, and within self-selected groups identified as having PAU, between 19 and 29% were of new onset. Using self-report questionnaires, new onset alcohol use (AU) ranged from 0–37%, and new onset AUD, SUD, AA or AD varied: short term 0.54–7% (n = 3 studies) medium term 3.8–8.5% (n = 4), long term 2.9–26% (n = 6). Most studies in the long term follow up groups had higher prevalence of new onset alcohol misuse (approaching or above 10%), compared to medium- and short-term studies. Stricter diagnostic criteria (e.g. AA or AD) tended to confer with lower prevalence rates compared to more inclusive criteria such as PAU or SUD. New onset AUD was most consistently reported in the studies and varied from 7.2–20.8% in medium- and long-term studies.

The studies measuring alcohol related ICD codes in hospital or bariatric cohorts and bariatric surgery prevalence in substance misuse programme attendees (n = 9) only considered long term. Kim et al. [34] found evidence of increased risk of new onset AA or AD in bariatric compared to cholecystectomy patients (AHR 2.7% vs 1.9%). Bramming et al. [22] found increased risk of new onset AUD in RYGB compared to age and weight matched controls at 5 years of 3.7% vs 0.8% controls and 10 years absolute risk 7.8% vs 1.4%. Studies of substance abuse programmes found that within bariatric surgery patients who were over-represented in these programmes, a high number of these were of new onset (17–60%).

This review has confirmed new onset concerning alcohol use post-bariatic surgery, the risk of which increases over time. The highest prevalence seems to be after RYGB, but this review shows for the first time that SG is not risk free. This conclusion is supported by self-report and clinical interview data as well as some objective data from registry and hospital data bases.

### Potential Etiological Pathways

The observed increased prevalence of new onset alcohol misuse after bariatric surgery has multiple potential causes. Some theories that have been commonly expostulated as to the reason for the observed increased rates of alcohol misuse in RYGB patients may apply to new onset alcohol misuse also. The most convincing of these is the difference in pharmacokinetics following bariatric surgery, such that higher concentrations of blood alcohol are achieved quicker, resulting in greater potential for addiction [61]. Both RYGB and to a lesser degree SG have this effect on alcohol absorption, but not LAGB [62–64]. Furthermore, Engel et al. [61] found that not only were the pharmacokinetics altered, but that the rewarding properties of alcohol were affected in RYGB too. The differences in pharmacokinetics of alcohol between RYGB, SG and LAGB could go some way to explain the differences in prevalence of AUD and new onset AUD in these groups. Other proffered reasons include increased socialization after weight loss and exposure to alcohol, and the loss of food for emotional regulation resulting in compensatory alcohol use. Ivezaj et al. [65] hypothesised that changes in hormones released by the gut following RYGB may affect brain reward centres and taste preference, thereby altering the salience of the rewarding properties of alcohol. This area is still an area of ongoing investigation for example, one of the most significant hormonal changes after bariatric surgery, Glucagon-like peptide-1 (GLP-1) which goes up after both SG and RYGB is being studied for its potential role as a treatment for AUD [66]. Individuals with depressive disorders, those addicted to substances or alcohol, smokers and people living with obesity show overactivity in the limbic-hypothalamic-pituitary-adrenal axis, one of the body’s stress response mechanisms. One of the effects of this overactivity in obese individuals is lowering serotonin levels. Carbohydrates, in particular sugar, which temporarily increases serotonin activity and improve mood are craved, and eating becomes a means of achieving emotional regulation. The neural pathways implicated in this and in hedonic reward from food are the same pathways that are activated by drugs of addiction such as heroin, amphetamines, cocaine, alcohol and nicotine. Fowler et al. [30] found that those who reported pre-surgical problems with High-Sugar/Low-Fat foods and those high on the glycemic index (GI) were at a greater risk of new onset SUD after surgery, supporting a suggestion of alcohol replacing food. On the other hand, Wiedemann et al. [59] found that those with a past history of SUD were more likely to have a history of BED than those that developed a problem after surgery de novo. The ‘addiction transfer’ theory has however been largely discredited due to findings that alcohol dependency is independent of food intake in both humans and animal models and the hormones in question appear to decrease rather than increase alcohol preference in animal models [67]. Furthermore, as argued by Ivezaj et al. [65], the latency seen in the development of alcohol problems and the differences in risk of SUD between procedure types suggest that addiction transfer cannot offer the only explanation. A qualitative study examining patient perspectives on this suggest that loss of control plays a central role in patients experience of post operative AUD and was associated with negative cognitions and emotions, including feelings of guilt and shame, which resonated with feelings around weight pre-surgery [68].

### Strengths and Limitations

A strength of this systematic review is that it is the first to focus on new onset alcohol misuse after bariatric surgery; previous reviews have focused on prevalence of alcohol use or misuse after surgery, without examining that cohort that develops alcohol misuse de novo. This is an important distinction, as alcohol misuse that develops de novo is differentiated from a pre-existing condition that has not improved, or worsened after an intervention, but rather is a potentially life-threatening complication of bariatric surgery, with implications of risk management and how patients are counselled about surgery.

Furthermore our study incorporates several recent studies which have specifically examined the risk of AUD in SG. Whereas previous reviews have not had sufficient data to comment on the risk in SG, taken together, these newer studies indicate that whilst RYGB appears to carry the highest risk, SG also carries a risk of AUD and cannot therefore be considered a risk free option in this respect, especially in patients who carry other risk factors for AUD.

The study extracted data on new alcohol misuse from studies employing a wide range of different measures of alcohol use and misuse and a further strength of the study was that we were able to summarize the various diagnostic criteria of alcohol misuse in order compare results across methodologies and time scales to achieve a clear picture of new emerging alcohol misuse after bariatric surgery. The inclusion of studies with follow up periods exceeding 2 years provided a key insight into the longer-term risk of new onset alcohol misuse. Data extraction was complicated by the fact that many of the studies were not specifically designed to collect data on new onset alcohol misuse but we were able to extract this data in 23 of the 42 studies.

Our results indicate some limitations in the available data. Firstly, new onset alcohol misuse is not routinely measured, and our study suggests it should be, given the apparent emergence of this issue, especially in the medium to longer term, and the over-representation of people with new onset alcohol misuse in SUD treatment programs. Secondly, the majority of the studies used self-report questionnaires such as AUDIT or AUDIT-C without any clinical interview to confirm the diagnosis. The sensitivity and specificity of these questionnaires for diagnosis has limitations, and interpretation in the light of changing DSM IV and V criteria over the search period meant that we have included different diagnoses under the term alcohol misuse. Thirdly, self-reported alcohol intake may also be underreported by patients undergoing bariatric surgery due to fear by patients of being excluded from having surgery. This limits the interpretation of the findings. Furthermore, despite the new data, subgroup analysis was not possible, limiting generalization of our findings. In addition, there were no RCTs, hence we were unable to explore alcohol misuse following bariatric surgery versus weight-loss via other methods such as pharmacological treatments. Although we included long term studies, in view of the latency in development of alcohol misuse development, several studies did not proceed for long enough to give data on our outcomes of interest. Studies relying on hospital data are subject to the risk of misclassification and under-reporting of diagnoses.

### Comparisons with Other Studies

Our data builds upon that published by Kanji et al. [10], as part of their qualitative scoping review to assess surgical outcomes in those with a history of substance use or SUD^.^. Our study extends the conclusions to include more convincing data on a small subset of patients that develop new onset concerning alcohol use, and that this prevalence increases after longer periods. Furthermore, we include more recent data on the differences between surgeries, especially SG, previously assumed to be low risk in this respect in the absence of data.

## Conclusion

This systematic review provides support for the current consensus guidance suggesting patients should be informed of a small but significant risk of new onset alcohol misuse after bariatric surgery and also provides supporting evidence of the risk of relapse from previous AUD. Although our study confirmed an increased risk of AUD, studies included various measures which encompass a wide range of severity of alcohol misuse following mostly RYGB, but also SG surgery to include AA, AUD and AD, with the strongest evidence for this increased risk in the medium to long term. The findings of this study reinforce the need for patient counselling before and after bariatric surgery on the risk of AUD postoperatively. It is important that screening for AUD is routinely carried out post-operatively, ideally using the AUDIT questionnaire as a screening tool, followed up by individual assessment by a healthcare professional if the patient scores above 8, indicating possible harmful use of alcohol. Onward referral to appropriate alcohol services should be initiated if AUD is confirmed on clinical interview. This is especially relevant in the medium to long term, when patients are often discharged from bariatric team follow up. More research is needed to understand whether the risk of de novo alcohol misuse differs between surgery type. Future studies examining bariatric surgery outcomes should routinely include measures of alcohol misuse, using validated questionnaires, supported by clinical interview.

## Data Availability

No datasets were generated or analysed during the current study.

## Abbreviations

AD: Alcohol dependency
AU: Alcohol use
AUD: Alcohol use disorder
AUDIT: Alcohol use identification test
AUDIT AUC: Alcohol use identification test short version
BED: Binge eating disorder
BMI: Body mass index
BPD: Biliopancreatic diversion
BPD-DS: Biliopancreatic diversion with duodenal switch
DSM: Diagnostic and Statistical Manual of Mental Disorders
ICD: International Classification of Diseases
IQR: Interquartile range
GI: Glycaemic index
GLP -1: Glucagon like peptide 1
HR: High-risk alcohol use
ICROMS: Integrated quality criteria for review of multiple study designs
LAGB: Adjustable gastric banding surgery
MAST: Michigan Alcohol Screening Test
MR: Medium risk alcohol use
NBSR: National Bariatric Surgery Registry
OP: Out-patient
PAU: Self-reported problematic alcohol use
PPDB: Presumed problematic drinking behaviour
PSU: Self reported problematic substance use
RYGG: Roux en Y Gastric Bypass surgery
SCID: Structured clinical interview for DSM IV axis 1 disorders,
SD: Standard deviation
SEM: Standard error of the mean
SG: Sleeve gastrectomy
SUD: Substance use disorder,
SUD Rx: Substance use disorder treatment,
UK: United Kingdom
USA: United States of America
WHO: World Health Organisation
